# Objective method for assessment of reliability of particle tracing visualization in 4D FLOW MRI

**DOI:** 10.1186/1532-429X-15-S1-E29

**Published:** 2013-01-30

**Authors:** Patrick J de Koning, Rob J van der Geest, Jos J Westenberg

**Affiliations:** 1 Dept. of Radiology, Leiden University Medical Center, Leiden, Netherlands

## Background

Three-dimensional (3D) velocity-encoded MRI (4DFLOW) of the heart may be used for the assessment of systolic and diastolic function. Particle tracing is frequently used as a technique for visualization of 3D intra-cardiac flow patterns. As the obtained velocity data may suffer from imperfections in the acquisition technique, it is important to have a means for image quality control. Consistency of the 4D FLOW data with other CMR measurements is a means for internal data consistency and may serve as surrogate validation. The purpose of the study was to apply particle tracing in 4DFLOW MRI of the heart and to compare aortic flow measurements derived from particle tracing with an alternative validated MR analysis technique.

## Methods

Whole heart velocity-encoded MR was performed in 7 healthy subjects (age 40±15 years) on at 1.5T MR system (Philips Healthcare). A 3D isotropic dataset of 4.2×4.2×4.2mm3 was acquired using retrospective gating with 30 phases reconstructed and velocity sensitivity of 150cm/s in all directions. Aortic (AO) flow was quantified using retrospective valve tracking (RVT) as described previously [1]. Particle tracing visualization was performed using in-house developed software using the VTK ToolKit. A 4th order Runge-Kutta numerical integration technique was implemented using a time step of 10 ms. At the moment of end-diastole all voxels within the LV cavity were traced in 3D over three cardiac cycles. The particles entering the proximal AO were recorded to compute the cumulative contribution to the AO flow of the particles initially enclosed in the LV cavity. Assuming a properly closed mitral valve, the summation of particles entering the AO during systole of the first cardiac cycle should constitute the LV stroke volume and should correspond to the AO flow assessed by the RVT method. In addition, the cumulative flow of particles into the AO over multiple cardiac cycles enabled studying the amount of retained flow in the LV.

## Results

**Figure 1:**
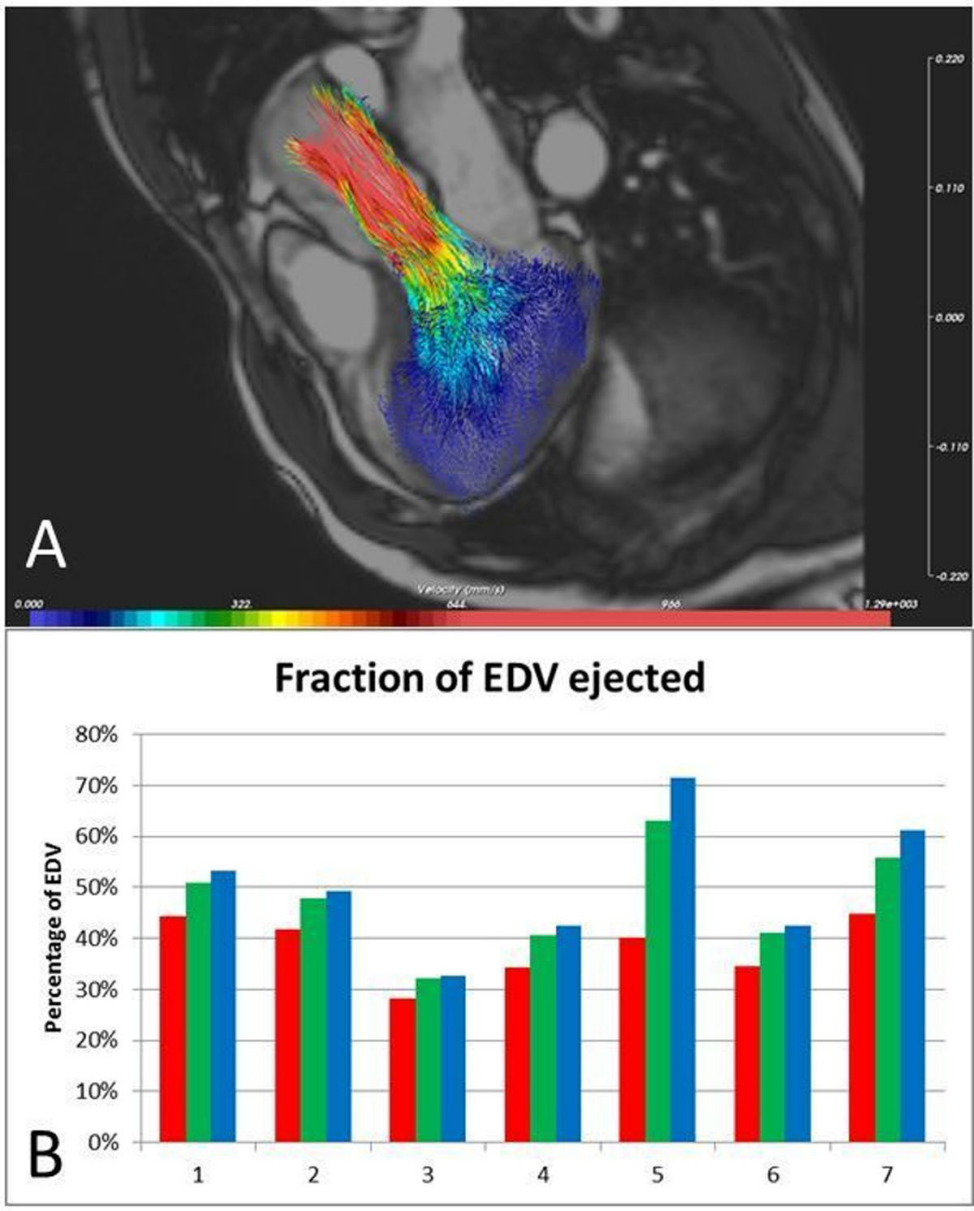
A. Example particle tracing visualization at systolic phase of first cardiac cycle. B. Percentage of LV blood particles released at the end-diastolic phase which has entered the aorta at end of first (red), second (green) and third (blue) cardiac cycle.

3D visualization of particle tracing provided realistic visualization of intra-cardiac flow over the cardiac cycle. Figure 1A shows an example of particle tracing visualization. The percentage of LV blood particles which have entered the aorta after the first, second and third cardiac cycle is depicted in Figure 1B. Table 1 presents AO flow measurements within the first cardiac cycle obtained by RVT and particle tracing. In all but 2 subjects AO flow derived from particle tracing was significantly under-estimated compared to RVT.

**Table 1:** Comparison of aortic flow assessed using either retrospective valve tracking method (RVT) or paticle tracing method.

Subject	Flow-RVT	Flow-Particle tracing	Difference (%)
1	72	73	-25
2	97	66	-25
3	91	63	-34
4	97	49	-35
5	83	69	0.6
6	69	70	-2.0
7	109	79	-30
Mean	88.4	67.0	-22.8
StDev	14.2	9.7	15.4

## Conclusions

Particle tracing visualization provides intuitive and realistic looking visualization of intra-cardiac flow patterns. However it may contain significant errors which may be attributed to noise and errors in the underlying MR velocity data. Checking internal consistence of trans-valvular flow with another established MR flow quantification method may be used as an objective method to assess the reliability of the provided visualization.

## Funding

Dutch Technology Foundation (STW): project number 11626.
